# Sample pooling as a strategy for community monitoring for SARS-CoV-2

**DOI:** 10.1038/s41598-021-82765-5

**Published:** 2021-02-04

**Authors:** Rafal Sawicki, Izabela Korona-Glowniak, Anastazja Boguszewska, Agnieszka Stec, Malgorzata Polz-Dacewicz

**Affiliations:** 1 Department of Biochemistry and Biotechnology, Medical University of Lublin, Chodzki 1, 20093 Lublin, Poland; 2 Department of Pharmaceutical Microbiology, Medical University of Lublin, 20-093 Lublin, Poland; 3 Department of Virology with SARS Laboratory, Medical University of Lublin, Lublin, Poland; 4 Department of Medical Microbiology, Medical University of Lublin, 20-093 Lublin, Poland

**Keywords:** Genetic testing, SARS-CoV-2

## Abstract

Sample pooling strategy was intended to determine the optimal parameters for group testing of pooled specimens for the detection of SARS-CoV-2 and process them without significant loss of test usability. Standard molecular diagnostic laboratory equipment, and commercially available centrifugal filters, RNA isolation kits and SARS Cov2 PCR tests were used. The basic idea was to combine and concentrate several samples to the maximal volume, which can be extracted with the single extraction column. Out of 16 tested pools, 12 were positive with cycle threshold (Ct) values within 0.5 and 3.01 Ct of the original individual specimens. The analysis of 112 specimens determined that 12 pools were positive, followed by identification of 6 positive individual specimens among the 112 tested. This testing was accomplished with the use of 16 extractions/PCR tests, resulting in saving of 96 reactions but adding the 40 centrifugal filters. The present study demonstrated that pool testing could detect even up to a single positive sample with Ct value as high as 34. According to the standard protocols, reagents and equipment, this pooling method can be applied easily in current clinical testing laboratories.

## Introduction

The coronavirus disease 2019 (COVID-19) pandemic has revealed the global concern of not only the ability to the appropriate management of COVID-19 disease, supporting the clinical decision-making process for infection control but also survey of large asymptomatic populations.

Given the limited testing capacity available, it remains uncertain whether the circulation of SARS-CoV-2 (Severe Acute Respiratory Syndrome Coronavirus 2) in the community precedes the identification of infected individuals. Rapid and accurate laboratory testing of SARS-CoV-2 is essential for early discovery, reporting, quarantine isolation, and cutting off epidemic transmission. Increasing of test number would enable tracking asymptomatic COVID-19 carriers and facilitating early detection of infection spread in the community. Particular importance is also to screen high-risk populations (such as healthcare personnel, nursing homes), to estimate the effectiveness of community measures and social distancing and monitor a safe return to work, schools and universities. The efficient and higher capacity of laboratory diagnostics is needed to support such efforts.

Due to extensively validated standard operating procedure, the World Health Organization (WHO) recommended molecular reverse transcription PCR (RT-qPCR) testing of respiratory tract samples as a method for the identification and laboratory confirmation of SARS-CoV-2 infection^1^. RT‐PCR assays targeting ORF 1a, ORF 1b, S gene, N gene of SARS‐CoV can detect < 10 genome copies/reaction^2,3^. However, limitations on testing capacity have restricted governments and institution's ability to measure community prevalence and minimise transmission.

In Poland, by November 14, 5,563,521 tests have been performed. The cumulative number of tests is estimated to be 635.5 per 1000 people. On November 13, the rolling 7-day average number of daily tests was 1.52 per 1000, while the WHO suggested around 10–30 tests per confirmed case as a general benchmark of adequate testing. The value in Poland is much lower and has had a declining tendency. The share of positive COVID-19 tests showed a sharply increasing tendency from September. In early September, this share was around 2.5%, but it approached 7.1% by October. On November 13, the 7-day rolling average of positive tests exceeded 44%. The WHO recommends that the share of positive tests should be kept below 5%. Therefore, mass testing is essential for a wide range of further COVID‐19 control strategies, including checking for and stopping community transmission.

Sample pooling is a strategy used for community monitoring of other infectious diseases such as trachoma, tuberculosis^4,5^. While several pooling approaches for SARS-CoV-2 detection were recently reported^6–10^, these studies mostly discussed theoretical considerations. This study demonstrated practical pooling solutions that save reagents and time by carrying out RNA extraction and RT-PCR on pooled samples. To analyzse the potential loss in sensitivity due to the dilution of weak positive specimens into pools of negative specimens, we also compared the cycle threshold (Ct).

## Results

### Sample size calculation

The hierarchical testing included an experimental prevalence rate of 5%, an assay lower limit of detection of 1 to 3 RNA copies/µL, an assay sensitivity of 97%, an assay specificity of 100%, a two-stage pooling algorithm, and a range of pool sizes of 3 to 20 samples. These calculations predicted the optimal testing configuration of initial pool size of 5, followed by individual testing. The expected number of tests for the optimal testing configuration is 2.10. That leads to an expected number of tests per individual of 2.10/5 = 0.42. Thus, two-stage hierarchical testing reduces the expected number of tests by 58% compared to individual testing. For the algorithm's overall implementation, the sensitivity is 0.9409, the specificity is 1.0000, the positive predictive value is 1.0000, and the negative predictive value was 0.9969.

### Sample pool strategy

Sample pooling strategy was intended to use standard molecular diagnostic laboratory equipment and commercially available RNA isolation kits and SARS Cov2 PCR tests. The pooling experiments were performed according to the manufacturer's instructions. The assumption was to pool multiple specimens and process them with the one RNA isolation and a single PCR test without significant loss of test sensitivity. The presented approach leads to effective cost drop up to sixfold per sample compared to individual patient testing. To test the effectiveness and sensitivity of that strategy, we performed two separate sets of experiments. In the first, one SARS CoV-2 positive sample was pooled with eight negative. In the second, one positive sample was merged with five SARS CoV-2 negative. The basic idea was to combine and concentrate several samples to the maximal volume, which can be extracted with the single QIAamp Mini column. All the swabs used in the project (both COVID 19 positive and negative) were processed (extracted and tested) individually to confirm their status. Also, all supernatants left after pooled samples concentrating were subject to further testing for SARS CoV-2 RNA presence, and no signal was detected.

**Figure 1:**
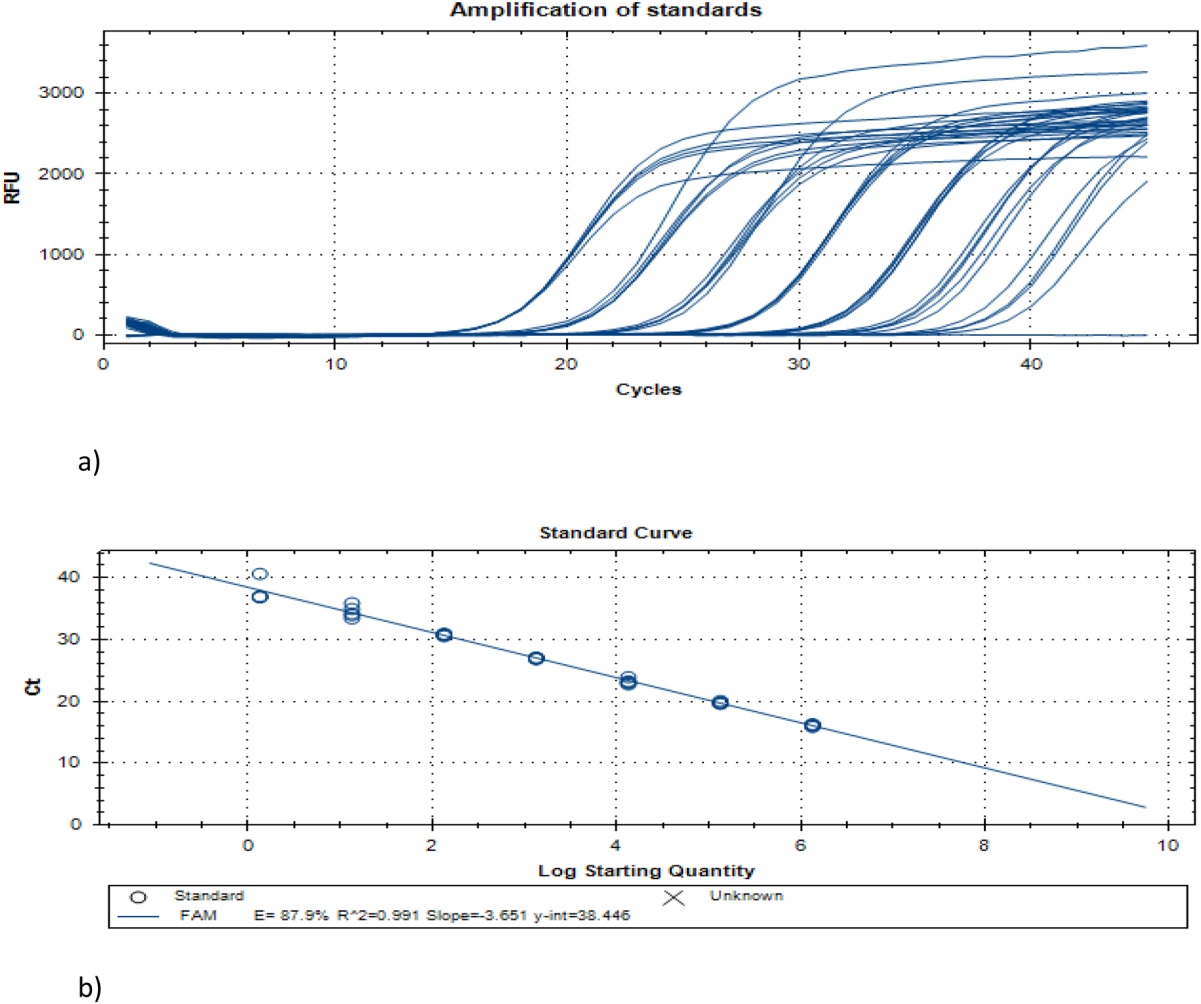
Amplification of standards*.* (**a**) Standards amplification curves; (**b**) Standard curve.

Conducted experiments showed the limits of sensitivity of the proposed strategy of pooling samples and Z-Path-COVID-19-CE Genesig Real-Time RT-PCR test itself. The extensive documentation prepared by the manufacturer was the reason for the test choice. The kit's instruction contains the information about analytical sensitivity and specificity as well as repeatability, inter-instrument reproducibility, operator reproducibility, daily reproducibility and accuracy. The declared effectiveness of SARS CoV-2 RNA detection with a 100% replicate detection at the level of about five virus copies per reaction was also confirmed in our research. Seven tenfold dilutions of standardised SARS-CoV-2 RNA templates starting from 1.336 × 10^6^ to 1.34 copies per reaction were amplified. The calculated standard curve showed excellent linearity r^2^ = 0.991, Fig. 1.

### Impact of pooling on the Ct value of the positive results (comparison of individual and pooled testing in parallel)

Obtained Ct values for pooled samples were compared with Ct values for SARS CoV-2 positive samples processed individually. The difference between these Ct's reflects the possible loss of initial viral RNA templates number in merged samples. Analytical Sensitivity Results demonstrates that the Coronavirus (COVID-19) CE IVD genesig^®^ kit detects 0.58 copies/µl of SARS-CoV-2 viral RNA with a confidence ≥ 95%. This concentration, therefore, serves as the limit of detection of the kit. Six and nine of pooled samples were tested in two different experiments in eight different reaction sets. Results were summarised in Tables 1 and 2.

**Table 1:** Six samples pooling tests results.

Sample name	Average Ct	∆Ct^a^	Calculated starting templates quantity	Copies/µl
Pooled group # 2	26.08 ± 0.26	1.41	2459.16 ± 407.82	307.4 ± 50.98
CoV-2 positive #2	24.68 ± 0.06		5921.69 ± 219.45	740.21 ± 27.43
Pooled group # 3	26.08 ± 0.65	1.25	2562.31 ± 898.64	320.29 ± 112.33
CoV-2 positive #3	24.83 ± 0.09		5383.47 ± 295.71	672.93 ± 36.96
Pooled group #1	30.34 ± 0.42	1.57	169.82 ± 42.64	21.22 ± 5.33
CoV-2 positive #1	28.78 ± 0.06		445.73 ± 16.31	55.72 ± 2.04
Pooled group # 8	31.58 ± 0.45	1.86	78.09 ± 22.88	9.76 ± 2.86
CoV-2 positive #8	29.72 ± 0.02		246.25 ± 2.70	30.78 ± 0.34
Pooled group # 4	31.36 ± 0.14	0.50	87.74 ± 7.68	10.97 ± 0.96
CoV-2 positive #4	30.85 ± 0.14		120.44 ± 10.41	15.06 ± 1.30
Pooled group # 5	35.14 ± 0.31	1.38	8.14 ± 1.56	1.02 ± 0.20
CoV-2 positive #5	33.76 ± 0.32		19.47 ± 4.06	2.43 ± 0.51
Pooled group # 7	–	–	–	–
CoV-2 positive #7	36.06 ± 0.16		4.52 ± 0.47	0.57 ± 0.06
Pooled group # 6	–	–	–	–
CoV-2 positive #6	36.13 ± 0.30		4.37 ± 0.79	0.55 ± 0.10
Average ∆Ct		1.33 ± 0.46		

^a^The difference between Ct values of the particular pooled group and corresponding positive sample.

**Table 2:** Nine samples pooling test results.

Sample name	Average Ct	∆Ct^a^	Calculated starting templates quantity	Copies/µl
Pooled group # 2	27.34 ± 0.24	2.62	1108.34 ± 164.61	138.54 ± 20.58
CoV-2 positive #2	24.72 ± 0.01		5749.46 ± 43.17	718.69 ± 5.40
Pooled group # 3	28.25 ± 0.37	2.59	630.24 ± 154.01	78.78 ± 19.25
CoV-2 positive #3	25.67 ± 0.05		3162.27 ± 92.19	395.28 ± 11.52
Pooled group #1	31.46 ± 0.31	1.80	83.05 ± 15.12	10.38 ± 1.89
CoV-2 positive #1	29.66 ± 0.16		256.22 ± 24.97	32.03 ± 3.12
Pooled group # 8	32.85 ± 0.24	2.85	34.31 ± 4.91	4.29 ± 0.61
CoV-2 positive #8	30.00 ± 0.14		205.49 ± 17.99	25.69 ± 2.25
Pooled group # 4	32.89 ± 0.14	2.59	33.36 ± 2.94	4.17 ± 0.37
CoV-2 positive #4	30.29 ± 0.18		171.58 ± 19.55	21.45 ± 2.44
Pooled group # 5	37.30 ± 1.72	3.01	2.69 ± 2.45	0.34 ± 0.31
CoV-2 positive #5	34.29 ± 0.37		13.99 ± 3.21	1.75 ± 0.40
Pooled group # 7	–	–	–	–
CoV-2 positive #7	35.71 ± 0.85		6.21 ± 3.60	0.78 ± 0.45
Pooled group # 6	–	–	–	–
CoV-2 positive #6	36.80 ± 0.06		2.83 ± 0.11	0.35 ± 0.01
	Average ∆Ct	2.58 ± 0.42		

^a^The difference between Ct values of the particular pooled group and corresponding positive sample.

Two of eight positive samples (#6 and #7) showed the Ct value below the Genesig Real-Time RT-PCR test guaranteed limit of detection, which was 0.34–0.55 viral copies per µl, (Tables 1, 2). However, both samples were analysed six times, and the repeatability of the test results was 100%. Positive status of these samples could not be confirmed with two other COVID 19 IVD multiplex tests (data not shown).

### Effect of number of samples in a pool

Calculated average delta Ct value, showed a statistical difference between pooled and individual positive samples in examined sets. The average 1.33 cycles difference in six samples pooling, confirmed the 2.5× loss of the initial amount of SARS CoV-2 RNA during the processing merged samples. The initial viral loss is even higher in nine samples pooling sets. The value of average delta Ct equal 2.58 can be translated to sixfold loss (5.97×) of the viral RNA through the pooling and concentrating process. The analytical sensitivity of the Genesig Real-Time RT-PCR test was equal to 4.64 SARS-CoV-2 RNA copies in reaction.

**Figure. 2:**
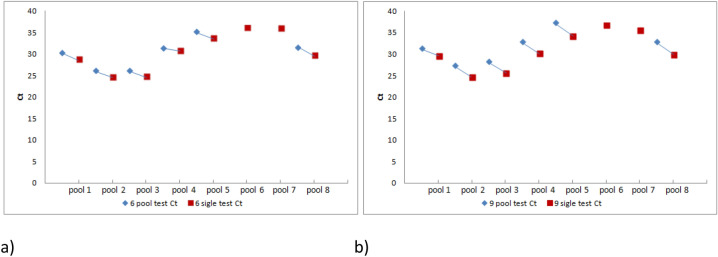
Ct values of single versus pooled samples. (**a**) 6 pooled sets; (**b**) 9 pooled sets.

Based on the test's sensitivity and confirmed loss of RNA during the concentrating process, the minimal initial viral RNA templates number that could be detected is 11.6 viral copies in the pooling of six samples, and 27.7 in nine samples merging. Many COVID 19 IVD multiplex real-time PCR test declare minimal analytical sensitivity on ten RNA copies in reaction. The Ct values for RdRP gene in both pools positive samples were positively detected if Ct values of single positive samples were up to 34 (Tables 1 and 2, Fig. 2). Pooling, concentrating and processing six samples in single RNA extraction followed by the SARS CoV-2 test, seemed to be an interesting alternative for lowering the already enormous COVID 19 molecular diagnostics costs.

## Discussion

This study demonstrates that specimen pooling can be easily provided in the laboratory to obtain faster and reliable test results. The primary and fundamental advantage of merging samples is the increase in availability and decrease in high-tech molecular diagnostics costs. The most crucial disadvantage is the loss of analytical sensitivity of the tests and a growing number of pooled samples. The strategy presented in this paper showed that an acceptable balance between the quantity of simultaneously tested samples and the analytical sensitivity of SARS CoV-2 testing could be reached.

Sample pooling is a strategy already used in sample screening for human immunodeficiency viruses (HIV)^11^, influenza^12^ as a simple, cost-effective method to enhance the speed of diagnosis especially when a large number of samples have to be tested during the epidemic screening^13^. The efficiency of pooling depends strongly on the prevalence of infection. Much recent literature has focused on pooling size selection for maximum efficiency based on prevalence^14–17^. Pooling is beneficial for routine community survey and monitoring of, essential workers, asymptomatic individuals and contact tracing. However, it is much less effective if used in settings with patients showing symptoms.

Due to the PCR test's high sensitiviy, the result should be positive if at least one of the samples is positive for SARS-CoV2. If the sample pool test is positive, each sample has to be re-tested individually. Recently, several research groups have proposed and developed testing protocols based on test pooling to make better use of the existing testing capacity^14,18,19^. For test pooling, patient's sample swabs are used twice: once in an archive tube and once in the pool tube. Several samples are combined in the pool tube so that they can be tested simultaneously.

In the present study, we randomly pooled the patient's samples in pools of 9 and 6, combined one positive sample in each pool, and we followed the protocols of RNA extraction and SARS CoV-2 PCR test. We added the additional step of samples concentration and used positive samples with a different load of viral RNA (Ct from 24.7 to 36.0) to validate the sample's method with a small amount of viral RNA. We found that in two pools, where low Ct positive samples (Ct > 35) were introduced, positive signal was lost. These samples were already beyond the test's guaranteed sensitivity limits during individual verification.

The 2.5-fold loss of the initial amount of SARS CoV-2 RNA, which can be translated to the average 1.33 cycles difference between pooled and single processed sample is the disadvantage of the method. Procedure processing of six samples in a single RNA extraction followed by the PCR appear to be equilibrium between lowering the test's costs and losing viral RNA template. In the time of the pandemic, this seems to be an acceptable price.

Three approaches to the pooling method can be applied: (i) collection of more than one swab to one sample with viral transport media; (ii) mixing of the samples from different patients before RNA extraction; (iii); separate RNA extraction from each sample and mixing many RNA isolates for one PCR test. Considering that, in the first option, when a pool turns out to be positive, repeat sample collection has to be done and that the third option is less economical, we chose the second option with some modification with specimen concentration. The similar pooling strategy approach, but without a sample concentration step, was presented by Abdulhamid et al.^19^ who tested one positive sample pool and four negatives where the maximum number of pooling of samples was assessed mathematically^19^. The study results showed that pooled specimens were positive within a range of 0 Ct to 5.03 Ct difference from the original samples. In our study, after samples were concentrated by centrifugation, the range of Ct differences was 0.5 to 3.01. Lim et al.^20^ described the pooled testing of 10 specimens starting from viral RNA extraction and followed by qRT-PCR while maintaining testing reliability. Although they did not perform any modelling on pool size before testing, they observed a threshold cycle (CT value) difference of less than three between an individual, and pooled testing and these findings are consistent with our and other studies^10^.

The third pooling option was described by Gupta et al.^21^ who recommended pooling of RNA samples before PCR as a faster and more economical method since otherwise when a pool turns out to be positive, repeat sample collection or RNA extraction has to be done^21^. Our results proved that the size of pooled specimens effectively diagnosed was equal to those pooled after RNA extraction the same being more cost-effective. In other published studies on pool testing the authors found that pooling of 1 positive sample up to a pool of 30^19^ or even 64 negative samples^10^ can be done without affecting the results. We have to remember that testing capacity of too large pool size will be wasted due to the possible need of re-testing (when too many pools are tested positive). It is essential to select a pool size that is optimal, in given the circumstances to make the best use of the existing testing capacity^22^.

In pooled strategy, there is a risk of missing the low positive cases as the viral load of low positive cases is diluted with increased volume in pooled samples. The authors^23,24^ explored the impact of dilution on the detectability of SARS-CoV-2 in asymptomatic patients using RT-PCR. Similarly to our results, they confirmed that the pooled approach with up to 5:1 specimen aliquots and using the current RT-PCR methodology would likely detect SARS-CoV-2 RNA among asymptomatic patients. We have seen that individual samples with borderline/low positive cases being positive in pooled samples showed Ct value 34. Garg et al.^25^ reported that pools with atypical/low positive cases showed Ct value above 30 (35.64–39.76), in most of the cases which on deconvoluted testing has lower Ct value below 30 (26.12–32.28). In another study on specimen pooling, it was observed that pooling did not affect the sensitivity of detecting SARS‐CoV‐2 when the PCR cycle threshold (Ct) of the original specimen was lower than 35^26^. However, Lohse et al.^19^ observed lower Ct values in some re-tested positive individual samples. They hypothesized that “the lower Ct values of pools than that of single samples were because of the carrier effect of the higher RNA content.” This is the major disadvantage of pooled testing. However, adding a few additional PCR cycles could be considered as a means to increase the detection rate of low-viral load samples^10^.

This study has some limitations. Firstly, a reference strain of SARS-CoV-2 was not included in the study and potential inhibitory effects of cellular material from pooling multiple clinical specimens and/or cross-reactivity of other respiratory viruses was not tested. Secondly, the presented method with the additional step of samples concentration by centrifugation before RNA extraction should be confirmed on the larger number of samples of pooled sets. Thirdly, the pooling approach works well when the disease prevalence is low. Currently, a very high level of Covid-19 in Poland was announced by CDC. Moreover, a strategy for fighting the pandemic in Poland is to continue with testing of symptomatic patients. Therefore, mass testing is important for a wide range of further COVID‐19 control strategies, including checking for and stopping community transmission.

## Materials and methods

### Sample collecting

The first step was to calculate the most efficient pool size according to the web-based application for pooling as described at https://www.chrisbilder.com/shiny, was used to assess the group testing strategy. At the Department of Virology with SARS Laboratory, Medical University of Lublin, Poland, samples from symptomatic patients, from the hospitals and samples from prospectively screened asymptomatic populations, such as hospital employees, and from the community were tested for SARS-CoV-2. In these samples, about 0.5% of SARS-CoV-2 tests were positive. According to the National Public Health guidelines, all samples were collected using a single swab for nasopharynx or combined deep nasal and oropharyngeal collection from the same patient. Nasopharyngeal swab samples were collected in 1 ml Sigma Virocult^®^ (Sigma-Aldrich). For this study, a total of 8 clinical specimens with a range of C_T_ values from 24 to 36 previously tested positive for SARS-CoV-2 by qRT-PCR and 104 negative patients swabs were selected.

### Samples concentration

The Amicon Ultra 0.5 ml Ultracell^®^ 30 K centrifugal filters (Merck) were utilizsed to concentrate the swabs samples. Three samples of 140 µl each were pipetted on a single filter. The obtained 420 µl of fluid was spun to obtain the desired final volume (40° fixed angle rotor, 14,000×*g*, room temperature). The exact time of centrifugation depended on the samples' viscosity, pooling test version and ranged from 10 to 25 min.

#### Nine samples pooling

**Figure 3:**
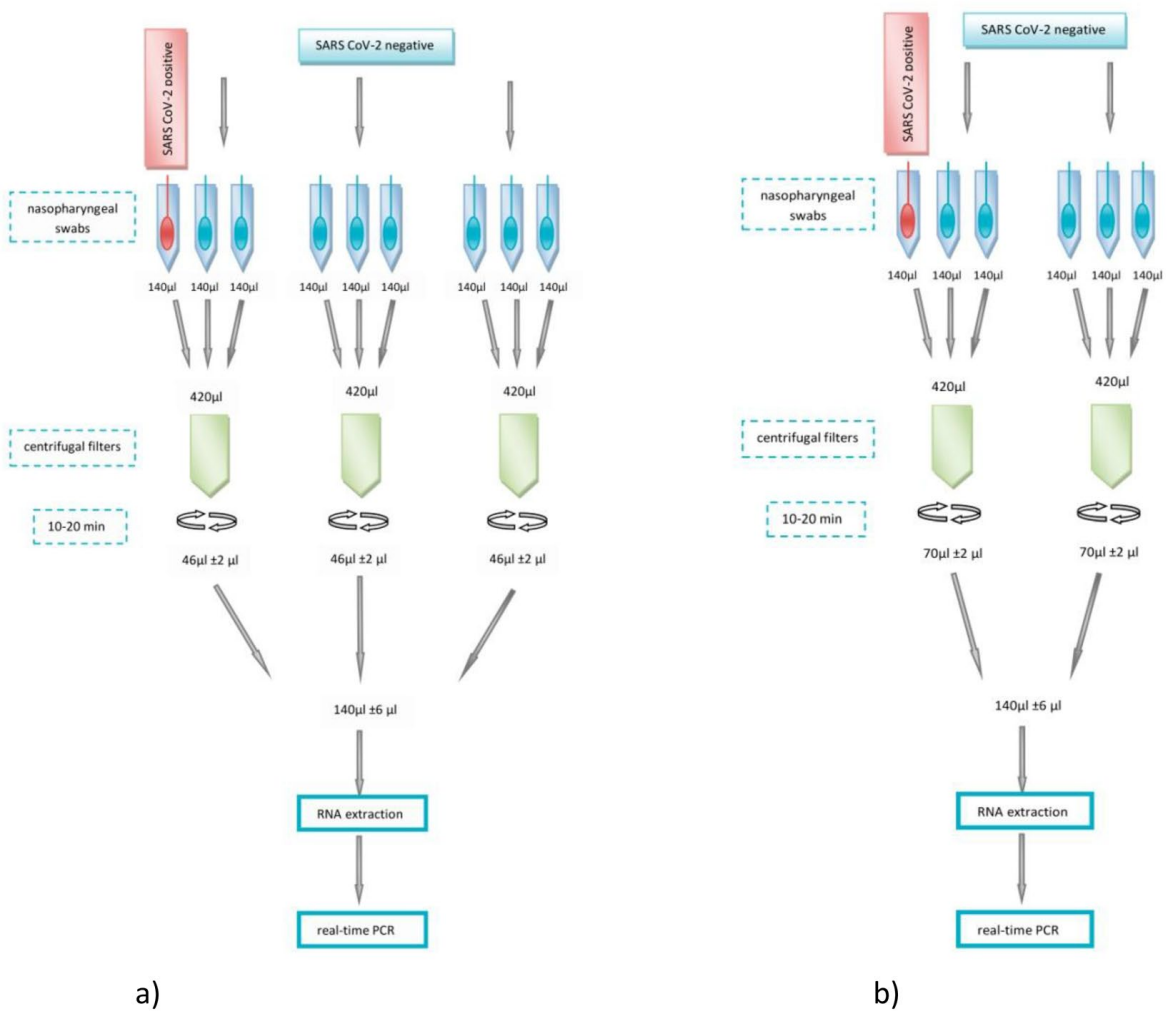
Samples pooling strategy. (**a**) Nine samples pooling; (**b**) Six samples pooling.

One positive and eight negative individual nasopharyngeal swab samples, each collected in 1 ml Sigma Virocult^®^, were first lysed under denaturing conditions, according to QIAamp Viral RNA Mini Handbook to inactivate RNases. The lysing buffer was replenished with the Genesig^®^Easy RNA Internal extraction control, which was a part of Coronavirus COVID-19 Genesig^®^Real-Time PCR assay. Then three Amicon Ultra 0.5 ml Ultracell^®^ 30 K centrifugal filters were loaded with nine samples. The first filer was filled with 140 µl of SARS CoV-2 positive material and two negative specimens 140 µl each. Two next Amicon filters were topped up with triplets of negative samples (140 µl each) to reach 420 µl of capacity in both concentrating tubes. The set of filters were centrifuged to obtain a final volume of 46 to 47 µl per filter. The samples concentrated on three separated Amicon filters were pooled. Received a volume of 140 µl was used to isolate viral RNA in the single isolation, Fig. 3a. The experiment was repeated on eight SARS CoV-2 positive and sixty-four SARS CoV-2 negative patient's swabs with three technical repeats.

#### Six samples pooling

The analogous procedure was adopted to concentrate and merge one SARS CoV-2 positive and five negative samples. Two Amicon Ultra 0.5 ml Ultracell^®^ 30 K centrifugal filters were loaded with 420 µl of three pooled samples per filter. The time of spinning the filters was selected to obtain about 70 µl of concentrated sample on each filter. Then samples were combined, and a total volume of 140 µl was subject to RNA extraction, Fig. 3b. The test was carried on eight SARS CoV-2 positive and forty SARS CoV-2 negative patient's swabs with three technical repeats.

### RNA extraction and RT-PCR testing

The RNA of eight SARS CoV-2 patients and one hundred and four SARS CoV-2 negative was extracted from Sigma Virocult^®^ swabs (140 µl per swab) using QIAamp Viral RNA Mini Kit (Qiagen) and eluted in 60 μL. The same protocol was adopted to isolate RNA from pooled samples. Eight μL of RNA was used in 20 μL reaction using Z-Path-COVID-19-CE Genesig Real-Time RT-PCR kit (Primerdesign). We followed kit instructions with thermocycler protocol: 1 cycle 55 °C 10 min; 1 cycle 95 °C 2 min; 45 cycles 95 °C 10 s; 60 °C 60 s. The quality of the RNA extraction step was verified by amplifying the Genesig^®^Easy RNA internal extraction control*. *Bio-Rad CFX Connect™ Real-Time PCR detection system was used to complete all real-time PCRs. The detection format was as follow: Covid-19 in FAM (465–510) channel, and RNA internal extraction control in HEX (533–580) channel.

### Amplification of standards and the standard curve calculation

The COVID-19 positive control template (PCT) was a part of the Z-Path-COVID-19-CE Genesig Real-Time RT-PCR kit and contained a standard number of copies SARS-CoV-2 RNA specific sequence with a given concentration of 1.67 × 10^5^ copies per μl (1.336 × 10^6^ copies in reaction). The PCT was employed to prepare a series of 7 tenfold dilutions and calculate the standard curve (Bio-Rad CFX Maestro software). The standard curve calculation was based on two separated experiments with five technical repeats per tested dilution.

### Ethics statement

The study was approved by the Ethics Committee review board of Medical University of Lublin, Poland (KE-0254/104/2020). The patient informed consent was waived off by the Ethical committee as the research was done on the anonymised, de-identified RNA samples. All methods were performed in accordance with the relevant guidelines and regulations.

## Conclusions

In our opinion, all ideas regarding SARS CoV-2 RNA pooling are worth developing to make the pandemic control more cost-effective. Moreover, this study showed that pooling approaches could facilitate patients testing during an infectious disease outbreak mainly by expanding current screening capacities of detection both in the community and among healthcare providers. During a rapidly changing epidemic, testing strategies will need to adapt to potential increases in positivity rate. However, the crucial challenge of pooled specimens testing is the use of highly sensitive assays to avoid missing low-positive samples. Therefore, laboratories must perform their validation pool studies for kits used for each RNA extraction and amplification based on the prevalence rate of COVID‐19 in their region.

## References

[1] Corman VM (2020). Detection of 2019 novel coronavirus (2019-nCoV) by real-time RT-PCR. Eurosurveillance.

[2] Emery SL (2004). Real-time reverse transcription-polymerase chain reaction assay for SARS-associated coronavirus. Emerg. Infect. Dis..

[3] Nitsche A, Schweiger B, Ellerbrok H, Niedrig M, Pauli G (2004). SARS coronavirus detection. Emerg. Infect. Dis..

[4] Ho J (2017). Pooling sputum samples to improve the feasibility of Xpert (R) MTB/RIF in systematic screening for tuberculosis. Int. J. Tuberc. Lung Dis..

[5] Ray KJ (2014). Estimating community prevalence of ocular chlamydia trachomatis infection using pooled polymerase chain reaction testing. Ophthalmic Epidemiol..

[6] Cleary B (2020). Efficient prevalence estimation and infected sample identification with group testing for SARS-CoV-2. medRxiv.

[7] Deckert A, Barnighausen T, Kyei NN (2020). Simulation of pooled-sample analysis strategies for COVID-19 mass testing. Bull. World Health Organ..

[8] Hogan CA, Sahoo MK, Pinsky BA (2020). Sample pooling as a strategy to detect community transmission of SARS-CoV-2. JAMA-J. Am. Med. Assoc..

[9] Shani-Narkiss H, Gilday OD, Yayon N, Landau ID (2020). Efficient and practical sample pooling for high-throughput PCR diagnosis of COVID-19. medRxiv.

[10] Yelin I (2020). Evaluation of COVID-19 RT-qPCR test in multi sample pools. Clin. Infect. Dis..

[11] Soroka SD, Granade TC, Phillips S, Parekh B (2003). The use of simple, rapid tests to detect antibodies to human immunodeficiency virus types 1 and 2 in pooled serum specimens. J. Clin. Virol..

[12] Fereidouni SR (2012). Saving resources: Avian influenza surveillance using pooled swab samples and reduced reaction volumes in real-time RT-PCR. J. Virol. Methods.

[13] Abel U, Schosser R, Suss J (1999). Estimating the prevalence of infectious agents using pooled samples: Biometrical considerations. Zbl Bakt-Int. J. Med. M.

[14] Abdalhamid B (2020). Assessment of specimen pooling to conserve SARS CoV-2 testing resources. Am. J. Clin. Pathol..

[15] Aragon-Caqueo D, Fernandez-Salinas J, Laroze D (2020). Optimization of group size in pool testing strategy for SARS-CoV-2: A simple mathematical model. J. Med. Virol..

[16] Bilder CR, Iwen PC, Abdalhamid B (2020). Pool size selection when testing for SARS-CoV-2. Clin. Infect. Dis..

[17] Cherif A, Grobe N, Wang X, Kotanko P (2020). Simulation of pool testing to identify patients with coronavirus disease 2019 under conditions of limited test availability. JAMA Netw. Open.

[18] Ben-Ami R (2020). Large-scale implementation of pooled RNA extraction and RT-PCR for SARS-CoV-2 detection. Clin. Microbiol. Infect..

[19] Lohse S (2020). Pooling of samples for testing for SARS-CoV-2 in asymptomatic people. Lancet Infect. Dis..

[20] Lim KL (2020). A novel strategy for community screening of SARS-CoV-2 (COVID-19): Sample pooling method. PLoS ONE.

[21] Gupta E (2020). Pooled RNA sample reverse transcriptase real time PCR assay for SARS CoV-2 infection: A reliable, faster and economical method. PLoS ONE.

[22] Klenske ED (2020). Optimal test pooling for efficient PCR testing of SARS-CoV2. Irish J. Med. Sci..

[23] Bateman AC, Mueller S, Guenther K, Shult P (2020). Assessing the dilution effect of specimen pooling on the sensitivity of SARS-CoV-2 PCR tests. J. Med. Virol..

[24] Smalley DL (2020). Impact of pool testing in detection of asymptomatic patients with COVID-19. Lab. Med..

[25] Garg J (2020). Evaluation of sample pooling for diagnosis of COVID-19 by real time-PCR: A resource-saving combat strategy. J. Med. Virol..

[26] Wacharapluesadee S (2020). Evaluating the efficiency of specimen pooling for PCR-based detection of COVID-19. J. Med. Virol..

